# Objective Markers for Diagnosing Concussions: Beyond Blood Biomarkers and the Role of Real-Time Diagnostic Tools

**DOI:** 10.3390/jcm14217727

**Published:** 2025-10-30

**Authors:** Robert Kamil, Youssef Atef AbdelAlim, Shiv Patel, Paxton Sweeney, Harry Feng, Jasdeep Hundal, Ira Goldstein

**Affiliations:** 1 Department of Neurosurgery, Hackensack University Medical Center, Hackensack, NJ 07601, USA; ira.goldstein@hmhn.org; 2 Department of Neurosurgery, University of Miami Miller School of Medicine, Miami, FL 07753, USA; 3 Department of Neurosurgery, Hackensack Meridian School of Medicine, Nutley, NJ 07110, USA; youssef.atefabdelalim@hmhn.org (Y.A.A.); shivy.patel@hmhn.org (S.P.); paxton.sweeney@hmhn.org (P.S.); harry.feng@hmhn.org (H.F.); 4 Department of Neurology, Jersey Shore University Medical Center, Neptune, NJ 07753, USA; jasdeep.hundal@hmhn.org

**Keywords:** concussion diagnosis, objective markers, neuroimaging, electrophysiology, EEG, biomarkers, SCAT5, real-time diagnostics

## Abstract

Concussions, classified as a type of mild traumatic brain injury (mTBI), are frequently underdiagnosed due to the subjective nature of symptoms and limitations in existing diagnostic methodologies. Current clinical evaluations, including tools such as the Sport Concussion Assessment Tool 5 (SCAT5), Balance Error Scoring System (BESS), and Vestibular Ocular Motor Screening (VOMS), demonstrate high sensitivity and specificity but often fail to capture the full complexity of concussive injuries. Emerging diagnostic approaches, such as blood biomarkers (for example, glial fibrillary acidic protein (GFAP), ubiquitin C-terminal hydrolase-L1 (UCH-L1), S100 calcium-binding protein B (S100B), and tau) and advanced neuroimaging techniques (for example, diffusion tensor imaging (DTI) and functional magnetic resonance imaging (fMRI)), show promise but remain impractical for routine clinical use due to accessibility and standardization challenges. This review examines objective markers, including neuroimaging, electrophysiological measures (for example, Electroencephalography (EEG), Magnetoencephalography (MEG)), and real-time diagnostic tools, as complementary strategies to enhance traditional clinical evaluations. Findings indicate that while clinical assessments remain central to concussion diagnosis, integrating them with advanced imaging and electrophysiological tools can provide more accurate diagnostics and recovery tracking. Biomarkers, although not yet ready for widespread use, hold significant potential for future applications. Further research is required to validate these methods and establish standardized protocols to facilitate their integration into clinical practice.

## 1. Introduction

Concussion, a subset of mild traumatic brain injuries (mTBIs), is defined as trauma-induced disturbances of brain function that are typically temporary [1]. Such injuries occupy the milder end of the traumatic brain injury (TBI) spectrum and are prevalent across various settings, including sports, military service, and civilian life [2]. The U.S. sees approximately 1.7 million TBIs annually [1,2], with estimates ranging up to 3.8 million when accounting for cases not requiring immediate medical care [1,2]. Sports-related activities are a significant contributor, accounting for an estimated 3.8 million concussions each year, with nearly half going unreported [3]. Military personnel, especially those deployed in conflict zones, also experience a high incidence of concussive TBIs, with more than 310,000 cases diagnosed between 2000 and 2014 [4].

Despite growing awareness, diagnosing concussions remains challenging due to their subjective nature and variability in symptom presentation [5]. The importance of accurate diagnosis is magnified by emerging evidence from longitudinal studies demonstrating the potential for subtle but persistent cognitive and neurological changes long after the initial injury [6]. The diagnostic complexity is underscored by recent consensus statements, such as the 2023 American Congress of Rehabilitation Medicine (ACRM) diagnostic criteria, which provide a unified framework for mild TBI across various settings [7]. Many patients do not show immediate symptoms and over 90% do not experience loss of consciousness [5,8], making clinical assessments difficult. Diagnosis often relies on tools like the Sport Concussion Assessment Tool 6 (SCAT6), Balance Error Scoring System (BESS), and Vestibular Ocular Motor Screening (VOMS) [9,10,11]. While these tools can be effective, their subjectivity and potential for inconsistent administration limit their ability to capture the full spectrum of concussive injuries [12,13].

The integration of objective markers, such as advanced neuroimaging techniques (for example, diffusion tensor imaging (DTI), functional magnetic resonance imaging (fMRI)) and electrophysiologic evaluations, including Electroencephalography (EEG), alongside clinical evaluations, presents an opportunity for more accurate and comprehensive assessments [5,8]. Although biomarkers like glial fibrillary acidic protein (GFAP) and ubiquitin C-terminal hydrolase-L1 (UCH-L1) hold promise, they are not yet practical for routine clinical use. This review also aims to explore the potential of combining clinical evaluations with objective markers, highlighting how these tools could improve concussion diagnosis and recovery tracking. The review also examines the role of real-time diagnostic tools in enhancing the precision of clinical outcomes.

## 2. Methods

This article is a structured narrative review designed to synthesize and integrate the current landscape of concussion diagnostics, from established clinical tools to emerging objective markers. A literature search was conducted using the PubMed (U.S. National Library of Medicine, Bethesda, MD, USA), Scopus (Elsevier B.V., Amsterdam, The Netherlands), and Google Scholar (Google LLC, Mountain View, CA, USA) databases to ensure a comprehensive and representative overview of the field.

The search strategy employed a combination of keywords, including “concussion,” “mild traumatic brain injury (mTBI),” “concussion diagnosis,” “biomarkers,” “GFAP,” “UCH-L1,” “neuroimaging,” “diffusion tensor imaging (DTI),” “functional MRI (fMRI),” “electroencephalography (EEG),” “SCAT5,” “real-time diagnostics,” and “wearable sensors.” This primary search was supplemented by a review of the reference lists from key articles and recent consensus statements.

Inclusion criteria for sources were peer-reviewed articles such as original research, systematic reviews, meta-analyses, and other narrative reviews, as well as consensus statements and clinical practice guidelines from major neurological and sports medicine organizations. All articles included were published in English. Foundational papers were included to provide essential context, while a specific focus was placed on literature published within the last three to five years to identify emerging trends and technologies. Exclusion criteria were non-peer-reviewed sources, such as conference abstracts or editorials without data. Studies focused exclusively on animal models, unless they provided foundational mechanistic insights, and case reports, unless they were uniquely illustrative of a key concept.

Transparency: Rigor, and Reproducibility Statement: This study adheres to the principles of transparency, rigor, and reproducibility as outlined by the *Journal of Brain Injury*. As a narrative review, a comprehensive literature search was conducted across multiple databases to ensure a representative synthesis of foundational and current findings in the field of concussion diagnostics. All methodologies were conducted following established protocols to ensure accuracy and reliability of results. The inclusion criteria for literature were pre-specified, and a comprehensive literature search was conducted across multiple databases to minimize bias and ensure a representative synthesis of findings.

Statistical Analyses: When applicable, analyses were performed using validated software, with detailed descriptions of each parameter provided in the manuscript to facilitate reproducibility. Relevant clinical tools, biomarkers, and neuroimaging technologies were evaluated using standardized and peer-reviewed frameworks, ensuring the replicability of results. Where data from external studies were incorporated, sources are cited explicitly, and data collection methodologies are referenced to enable independent verification. Furthermore, while this is a narrative review and not a systematic review, its structure was informed by the principles of the PRISMA (Preferred Reporting Items for Systematic Reviews and Meta-Analyses) guidelines to ensure methodological rigor and transparency throughout (Supplementary Materials). This manuscript does not include experimental datasets, as it is a review article; however, all referenced studies are publicly available. Future researchers are encouraged to use this synthesis as a foundation for prospective, reproducible studies. Efforts to enhance reproducibility include thorough documentation of the search strategy and critical assessment of potential biases in included studies. The limitations of this review are explicitly discussed to provide a balanced interpretation of findings and identify avenues for further research.

Drug(s)/Device(s) Statement: This manuscript submitted does not contain information about medical device(s)/drug(s).

## 3. Current Concussion Evaluation and Diagnostic Methodologies

The diagnosis of concussion relies on a combination of clinical tools, each offering unique insights into a patient’s condition. One of the most widely used methods is the Sport Concussion Assessment Tool 5 (SCAT5), developed by the Concussion in Sport Group. SCAT5 employs a stepwise evaluation approach that covers immediate red flags, observable symptoms, cognitive function, and neurological assessment [14]. This includes screening for red flags, such as loss of consciousness or seizure, cognitive assessments for memory and orientation, and balance tests through neurological evaluations [15]. Despite its widespread use in sports, SCAT5 is not without drawbacks. It shows high sensitivity (100% within 95% CI of 88.1–100) but its specificity remains low at 20% (95% CI 5.7–43.7), potentially leading to false positives [16]. The test’s 21 min duration also increases the likelihood of interruptions, which can affect diagnostic accuracy [16].

Another critical tool is the BESS, which is specifically designed to assess postural stability in concussed patients [17]. The BESS involves three different stance evaluations—single-leg, tandem, and double-leg stances—to gauge balance under varied conditions [18]. It can be completed in about five minutes, making it a practical option in many clinical settings. The BESS, however, also has a mixed sensitivity range (34–64%) but boasts a specificity of 91%, emphasizing its strength in ruling out non-concussed patients while potentially missing some cases [19].

The VOMS tool complements other diagnostic approaches by focusing on vestibular and oculomotor impairments following a concussion [20]. VOMS tests include assessments of smooth pursuits, saccades, vestibulo-ocular reflexes, near-point convergence, and visual motion sensitivity [20]. While VOMS is highly effective in identifying concussion-related impairments that other tools might miss, its sensitivity (58–96%) and specificity (46–92%) vary widely [13], making it best used alongside other diagnostic measures for a more comprehensive assessment. The application and interpretation of these tools are particularly critical in pediatric and adolescent populations, where recent studies and guidelines highlight differences in symptom presentation and recovery trajectories compared to adults [21,22].

Beyond clinical tools, biomarkers are emerging as promising indicators for concussion diagnosis, especially for detecting subtle physiological changes not visible through standard evaluations. Biomarkers such as GFAP, UCH-L1, S100 calcium-binding protein B (S100B), and tau are increasingly used to assess neuronal, axonal, and astroglial damage in the brain [23,24]. These biomarkers can help reduce the need for unnecessary head CT scans by offering a more targeted approach to identifying brain injuries [23,24,25]. Biomarker levels typically increase following a traumatic brain injury, providing clinicians with objective data to inform their diagnosis and predict patient outcomes [26].

Despite their promise, blood-based biomarkers face challenges that limit their utility in real-time clinical settings. Assays often take hours to yield results, making them less effective in emergency care scenarios where timely decisions are critical [27]. Variations in baseline biomarker levels between individuals can also complicate the interpretation of results, and most tests tend to have low specificity despite high sensitivity [28]. More research is needed to standardize their use in routine clinical practice and enhance their accuracy. Furthermore, emerging ultrasensitive immunoassay technologies, such as Single Molecule Array (Simoa), show potential for measuring key protein biomarkers like tau and neurofilament light chain (NfL) in blood, plasma, or cerebrospinal fluid (CSF) at much lower concentrations than previously possible, which may improve diagnostic sensitivity, particularly in the subacute phase of injury.

The integration of biomarkers with traditional clinical evaluation tools presents an opportunity to improve concussion diagnostics, particularly in scenarios where patients underreport symptoms or where symptoms are less apparent [29]. This approach is especially beneficial for athletes and individuals who may return to physical activities or daily routines before fully recovering from a concussion. By incorporating biomarkers, clinicians can access objective data that complement the subjective assessments of tools like SCAT5, BESS, and VOMS, leading to more precise and individualized care. This combined strategy not only enhances the accuracy of concussion diagnosis but also offers a clearer path for monitoring recovery, ultimately helping patients achieve better long-term outcomes [29].

## 4. Objective Markers for Concussion Diagnosis

Diagnosing concussions involves a range of advanced neuroimaging techniques, electrophysiological measures, and other emerging diagnostic technologies that offer more objective insights into brain function and injury.

Among these techniques, three key neuroimaging methods stand out: DTI, fMRI, and Magnetic Resonance Spectroscopy (MRS). DTI is a specialized form of MRI that evaluates the integrity of white matter by tracking the movement of water molecules along neural pathways. This technique can reveal microstructural changes in the brain that are often invisible with standard imaging methods. DTI provides quantitative metrics like fractional anisotropy (FA) and mean diffusivity (MD), which measure the directionality and movement of water molecules. In cases of white matter injury, such as concussions, FA values typically decrease, while MD increases, indicating disrupted neural pathways [30]. This technique also allows for the generation of 3D models of white matter pathways, known as tractography, which can visualize disruptions to specific neural tracts and may aid in detecting subtle microhemorrhages.

Functional MRI assesses changes in blood flow using blood-oxygen-level-dependent (BOLD) signals as a proxy for brain activity. This method can detect changes in neuronal function even when structural damage is not evident. Additionally, fMRI enables analysis of functional connectivity by examining synchronization between different brain regions [31]. MRS further enhances concussion diagnosis by detecting specific brain metabolites like N-acetylaspartate (NAA), creatine (Cr), choline (Cho), and lactate. A decline in NAA suggests decreased neuronal health, while elevated choline levels may indicate membrane damage. MRS often measures these metabolites in relation to creatine levels to differentiate between localized and diffuse brain injury [32]. Furthermore, recent systematic reviews highlight that advanced analytical techniques, such as resting-state functional connectivity and network analysis, are increasingly able to detect subtle disruptions in large-scale brain networks post-concussion, serving as valuable research tools even when not yet recommended for routine clinical use [33,34].

Complementing neuroimaging techniques, electrophysiological studies provide a different perspective by focusing on the brain’s electrical activity. Unlike neuroimaging, which reveals structural and metabolic changes, electrophysiological methods like EEG analyze the brain’s electrical patterns through electrodes placed on the scalp. This technique helps identify abnormal brainwave patterns, such as disrupted alpha or theta waves, which are often seen after a concussion. EEG also uses power spectral density (PSD) to measure the brain’s electrical power across various frequency bands, revealing shifts from higher to lower frequency bands after injury. Other EEG-based measures include coherence, which assesses functional connectivity, and event-related desynchronization (ERD), a marker of changes in brain rhythm [35].

Event-related potential (ERP) analysis is another valuable tool, providing insight into how quickly the brain processes auditory and visual stimuli. ERPs involve recording brain responses to specific stimuli, such as sounds or images, using electrodes placed on the scalp. Two key ERP components, P300 and N200 waves, reflect different cognitive processes. P300 waves are associated with attention and decision-making, while N200 waves are related to conflict detection and response inhibition. Both are often delayed or diminished in individuals with concussions, offering important markers for cognitive impairment [36]. Magnetoencephalography (MEG) measures the magnetic fields generated by brain activity, offering precise localization of functional disruptions. MEG can assess connectivity between brain regions, which is typically reduced after a concussion, and identify the source of abnormal signals through techniques like source localization. It also examines disruptions in neural oscillations across delta, theta, alpha, and beta wave bands, providing further insight into the functional impact of a concussion [37].

Beyond these established methods, new technologies like Near-Infrared Spectroscopy (NIRS) and Balance and Gait Analysis Systems are being explored for their potential in concussion diagnosis. NIRS measures cerebral oxygenation and blood flow through infrared light emitted from optodes placed on the scalp. This light penetrates the skull, detecting hemoglobin absorption levels to quantify both oxyhemoglobin (HbO) and deoxyhemoglobin (HbR). Variations in these levels reflect hemodynamic changes in the brain following a concussion. Analyzing the relationship between HbO and HbR also provides insights into oxidative metabolism, which is often reduced after a concussive event.

## 5. Real-Time Diagnostic Tools for Concussion

Recent advances in portable EEG devices have shown potential for real-time concussion diagnosis, offering an alternative to traditional EEG methods. Conventional EEG monitoring requires extensive preparation, including the use of conductive gels, and is typically confined to laboratory settings, limiting its practicality in diagnosing acute concussions [38]. This has led to the exploration of portable EEG systems suitable for use in diverse settings like homes, sporting events, and public places. These newer devices utilize dry sensors, single-channel, and frontal electrode recording, eliminating the need for gels and reducing setup complexity. A study by Fronso et al., (2019) comparing a 64-channel dry electrode cap to a traditional gel-based system found that preparation time for the dry cap was significantly shorter—13 ± 3 min compared to 39 ± 18 min for the gel-based system [39]. Despite the reduced setup time, the study also highlighted reliability differences. During resting phases, the gel-based system achieved 95 ± 3% channel reliability compared to 80 ± 15% for the dry cap. During active movement, reliability decreased for both systems but remained higher for the gel-based contacts (85 ± 9%) compared to the dry cap (66 ± 19%). These findings suggest that while portable EEG offers practical advantages in terms of speed, it may sacrifice some reliability, particularly during physical activity.

Beyond EEG, the potential of bedside neuroimaging for real-time concussion diagnosis is also being explored. Portable MRIs, designed for use at the bedside, operate without cryogens, connect to standard power outlets, and utilize lower magnetic fields (0.064T versus the conventional 1.5–3T) [40]. A point-of-care study by Sheth et al., (2020) [40] evaluated the performance of portable MRI in diagnosing various brain injuries, including TBI. The results demonstrated that portable MRI findings matched conventional radiology reports, except in one case where a diffuse subarachnoid hemorrhage was missed. These findings suggest that portable MRI could be a valuable tool in the acute diagnosis of concussions, warranting further exploration.

Portable CT scanners are also valuable for assessing head injuries, particularly where access to traditional imaging is limited. While concussions often lack detectable abnormalities on standard CT scans, these mobile devices are instrumental in identifying more severe intracranial injuries like hemorrhages or fractures that may accompany a concussion. Their rapid, on-site evaluation facilitates timely medical intervention. It remains critical to recognize, however, that a negative CT scan does not rule out a concussion, as these injuries can occur without visible structural damage. A negative scan is primarily a tool to identify acute, life-threatening injuries, thereby contextualizing the diagnosis of mTBI within the broader spectrum of head trauma [41]. Therefore, clinical assessment remains paramount in the diagnosis and management of concussions.

Eye-tracking technology is another promising non-invasive method for real-time concussion assessment. It evaluates gaze patterns, pupil diameter fluctuations, and spontaneous blink rates to assess attention, cognitive function, and neurological activity. These metrics provide insights into adrenergic and cholinergic function, making eye tracking a useful tool in studying mental fatigue and task disassociation [42,43]. Its application in concussion diagnosis is still being refined, as studies face challenges like population heterogeneity and varying diagnostic criteria [44]. A prospective study by Samadani et al., (2022) in an emergency room setting found that their eye-tracking algorithm achieved 80.4% sensitivity and 66.1% specificity, with an AUC of 0.718. A significant misclassification rate (31.6%), however, highlighted the impact of patient diversity on diagnostic accuracy [44]. Despite these limitations, eye-tracking has shown promise in post-concussion assessments. For instance, a study by Zahid et al., (2020) focusing on children found that certain eye-tracking metrics, including near-point convergence (NPC) disability, provided 95.8% specificity and 57.1% sensitivity for concussion detection (AUC = 0.810), suggesting its potential for detecting CNS changes after injury [45]. Further test-accuracy studies are needed to fully evaluate the ability of these systems to diagnose mTBI and predict protracted recovery [46]. In addition to eye-tracking, other automated optical tracking devices have been employed to evaluate posture and balance, integrating tools such as force plates. These devices, potentially augmented by artificial intelligence, show promise in identifying balance disorders and enhancing concussion diagnostics, particularly in diverse clinical settings [47].

Wearable technology equipped with sensors for monitoring balance, gait, and cognitive function has also emerged as a valuable tool in concussion diagnosis. These devices offer real-time data on motor control and cognitive deficits that may not be easily detected through standard clinical assessments [48]. Studies have shown that wearable sensors can identify subtle changes in gait and balance following a concussion [49], as well as assess cognitive functions like reaction time and memory recall [50]. This capability makes wearables particularly useful for guiding return-to-play decisions in athletes, helping to prevent premature activity resumption and the risk of prolonged neurological issues [50,51]. Wearable technology provides a non-invasive, cost-effective solution for continuous monitoring and concussion diagnosis in both athletic and non-athletic populations [51].

In summary, portable EEG devices, bedside neuroimaging, eye-tracking technology, and wearable sensors each offer unique advantages for real-time concussion diagnosis. Portable EEG provides quicker setup but faces challenges with reliability during movement [39]. Bedside MRIs hold promise for accurate acute diagnosis in various brain injuries, including concussions [40]. Eye-tracking, despite some current limitations, offers valuable insights into cognitive dysfunction, particularly in post-concussion recovery [45]. Meanwhile, wearable technology supports continuous monitoring of motor and cognitive functions, offering a comprehensive approach to managing concussions [48]. Integrating multimodal evaluation—including tools like force plates and automated tracking systems—can also help to minimize misdiagnosis of balance disorders, such as vertigo, as cases of concussion. Together, these tools represent a promising future for improving the precision and accessibility of concussion diagnostics.

## 6. Comparison of Objective Concussion Markers with Blood Biomarkers

The effectiveness of different concussion diagnostic tools varies significantly, depending on the type of tool used and the timing of the assessment. A summary of these tools, including their sensitivity, specificity, and practical considerations, is provided in Table 1. Traditional tools like the SCAT5, King-Devick, and ImPACT have been well-researched and are widely used. These methods exhibit relatively high sensitivity for diagnosing acute concussions, ranging from 0.80 to 0.88, but their specificity varies more, from 0.50 to 0.85 [52]. In contrast, portable EEG devices and eye-tracking tools, which measure real-time neural activity and oculomotor changes, tend to have lower sensitivity (0.48–0.58) and specificity (0.52–0.61). This reduction in accuracy is often due to external environmental factors and movement, which can introduce variability [53]. Blood biomarkers like GFAP and UCH-L1 have been validated for acute concussion diagnosis, showing higher sensitivity (0.84–0.94) within the first 6 h post-injury, but their reliability diminishes as time progresses [23]. The field is now moving beyond single markers, with recent studies showing that a panel combining multiple biomarkers, such as GFAP and NfL, significantly improves prognostic accuracy for patient outcomes [54]. The variability in sensitivity and specificity among these tools indicates that a combined approach using multiple diagnostic methods may yield a more comprehensive evaluation of concussion.

The timing of using specific diagnostic tools is crucial, as their effectiveness can change significantly depending on the stage of the injury. For example, salivary microRNAs (miRNAs) peak within 24–72 h post-injury and can differentiate concussed from non-concussed individuals with a specificity of up to 0.81 [55]. Recent research has further advanced this area, demonstrating novel platforms for the rapid, non-invasive detection of specific mTBI-associated salivary microRNAs with high sensitivity and specificity [56]. Blood biomarkers such as S100B and UCH-L1 are most reliable within the first 48 h but lose effectiveness beyond 72 h due to declining levels [23]. Neurocognitive tools like ImPACT are better suited for assessing longer-term cognitive effects of concussions, maintaining moderate sensitivity (0.62) even weeks after the initial injury [57]. Real-time tools like EEG are effective at detecting acute changes in brainwave activity immediately after the injury, but their diagnostic value decreases during the recovery phase. Understanding these temporal dynamics is essential for clinicians when selecting the appropriate diagnostic tools based on both the timing of the injury and the clinical presentation.

While the sensitivity and specificity of diagnostic tools are key considerations, their practicality in real-world settings is also critical. Tools like SCAT5 and King-Devick, which require minimal equipment and can be administered on the sidelines, are favored in sports settings for their convenience, though they may not be sensitive enough to detect subclinical concussions [52]. In contrast, portable neuroimaging and EEG devices provide more detailed assessments but are often limited by their higher costs, the need for trained personnel, and reduced reliability outside controlled environments [53]. Blood biomarkers hold promise in clinical and hospital settings but are less suitable for on-field use due to the requirement for laboratory analysis. Salivary biomarkers, being non-invasive, offer a potential alternative, yet they require further validation and standardization before being fully integrated into clinical practice [55,58]. The ultimate choice of diagnostic tool should balance accuracy, timing, and practicality, considering the specific clinical context in which the concussion evaluation occurs.

## 7. Future Directions in Concussion Diagnosis

The field of concussion diagnosis is evolving, with emerging technologies poised to enhance current methods. Among these innovations are novel diagnostic tools, such as advanced wearable devices and AI-driven diagnostics, that aim to provide more accurate and real-time assessment of concussive injuries. Wearable devices can continuously monitor key physiological parameters, offering insights into subtle changes that may indicate a concussion, while AI algorithms can analyze complex data patterns from these devices—for example, subtle changes in gait, heart rate variability, or electrophysiological signals—to deliver precise diagnostic outcomes. For instance, recent work has demonstrated that Bayesian machine learning models can use connectomic data to distinguish concussed individuals from healthy controls with high accuracy [59]. These technologies represent a shift toward more accessible, user-friendly, and real-time diagnostic capabilities.

**Figure 1:**
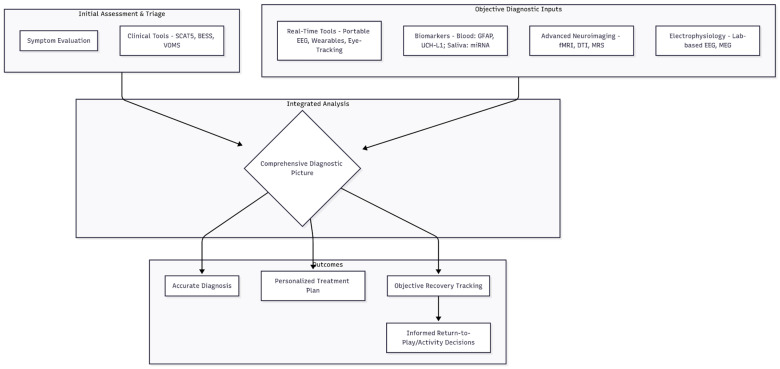
A Multimodal Integration Model for Concussion Diagnosis and Management. This diagram shows a multimodal approach where initial clinical assessments are combined with a suite of objective diagnostic inputs. These data are integrated to form a comprehensive diagnostic picture, which in turn informs an accurate diagnosis, personalized treatment, objective recovery tracking, and safer return-to-activity decisions.

A promising direction is the adoption of multimodal approaches, which integrate various diagnostic methodologies to create a more comprehensive understanding of concussions. This integrated framework is visualized in Figure 1. By combining neuroimaging techniques, electrophysiological assessments like EEG, and established clinical evaluations, healthcare providers can achieve a multi-dimensional view of a patient’s condition. This integration allows for a more nuanced understanding of the nature and severity of concussions, thereby enabling better-targeted treatment plans and more accurate monitoring of recovery progress. The convergence of these methods holds the potential to bridge existing diagnostic gaps.

While promising, several research gaps and challenges remain. There is a pressing need for further validation of non-invasive objective markers to ensure their reliability and accuracy across diverse populations. The development of standardized diagnostic protocols is also essential to harmonize the use of these emerging tools in clinical practice. Establishing clear guidelines and evidence-based practices would not only enhance the consistency of concussion diagnoses but also improve the quality of care provided to patients across different settings.

## 8. Ethical and Logistical Considerations for Real-Time Monitoring

The implementation of real-time monitoring technologies, such as wearables and AI-driven diagnostics, introduces important ethical and logistical considerations. From an ethical standpoint, patient privacy and data security are paramount. Continuous collection of sensitive physiological and neurological data requires robust encryption, secure storage, and clear policies regarding data ownership and access to prevent misuse or breaches. Logistical hurdles include cost, the need for user training, and ensuring equitable access across different socioeconomic and geographic settings. A final consideration is the potential for data misinterpretation or over-reliance on technology without clinical correlation, which must be addressed through rigorous validation and the development of clear clinical integration guidelines.

## 9. Conclusions

The accurate diagnosis of concussions requires a multifaceted approach that combines traditional clinical evaluations with advanced, real-time, and non-invasive diagnostic tools. The integration of neuroimaging, electrophysiological measures, and innovative wearable technology can provide a more precise understanding of concussion-related changes in the brain. This combined approach is crucial for capturing the full scope of concussive injuries, improving diagnostic accuracy, and ensuring a comprehensive evaluation of each patient’s condition.

The clinical implications of this integrative approach are significant. By adopting advanced diagnostic tools alongside conventional methods, clinicians can enhance their ability to diagnose concussions more accurately and implement tailored treatment strategies, which ultimately improves patient outcomes. This is particularly critical in scenarios where symptoms may be unclear or when monitoring recovery over time is essential. With the increasing scrutiny of concussions in scholastic and professional sports, as well as heightened concerns surrounding return-to-play decisions following head impacts, the demand for accurate and rapid on-site concussion assessment continues to grow. Standardizing diagnostic protocols and leveraging these innovative technologies, guided by recent evidence-based guidelines for graduated return-to-play protocols, will not only improve safety but also foster confidence among participants in contact sports.

## Figures and Tables

**Table 1:** Summary of Concussion Diagnostic Tools.

Tool/Marker	Description	Sensitivity	Specificity	Practicality & Notes
**SCAT5**	Stepwise clinical evaluation of symptoms, cognition, and neurological function.	High (up to 100%)	Low (~20%)	Widely used, sideline-friendly. Long duration (~21 min) can lead to interruptions. Low specificity can cause false positives.
**BESS**	Assesses postural stability through three different stances (single-leg, tandem, double-leg).	Mixed (34–64%)	High (91%)	Quick to administer (~5 min). Good for ruling out concussion but may miss cases due to mixed sensitivity.
**VOMS**	Screens for vestibular and oculomotor impairments (smooth pursuits, saccades).	High (58–96%)	Mixed (46–92%)	Identifies impairments other tools may miss. Best used as part of a comprehensive assessment due to wide variability.
**Blood Biomarkers (GFAP, UCH-L1)**	Measures proteins released into the blood following brain injury.	High (84–94%) within 6 h	Variable	Most reliable in the acute phase (<72 h). Can reduce need for CT scans. Lab analysis required, limiting on-field use.
**Portable EEG**	Measures brain electrical activity using dry or single-channel sensors.	Lower (48–58%)	Lower (52–61%)	Fast setup, highly portable. Sacrifices some reliability compared to lab-based EEG, especially during movement.
**Eye-Tracking**	Non-invasively assesses gaze patterns, pupil response, and blink rates.	Moderate (~80%)	Moderate (~66%)	Promising for objective cognitive assessment. Accuracy can be affected by population diversity. Useful in post-concussion recovery.
**Wearable Sensors**	Devices that monitor balance, gait, and cognitive function in real-time.	Variable	Variable	Provides continuous, objective data. Useful for return-to-play decisions. Requires further validation and standardization.

## Data Availability

No new data were created or analyzed in this study.

## Supplementary Materials

The following supporting information can be downloaded at: https://www.mdpi.com/article/10.3390/jcm14217727/s1, Table S1: PRISMA 2020 Checklist.
