# Supplementary material for: Objective Markers for Diagnosing Concussions: Beyond Blood Biomarkers and the Role of Real-Time Diagnostic Tools
Source: J Clin Med. 2025 Oct 30;14(21):7727. doi: 10.3390/jcm14217727 (PMC12609333; doi:10.3390/jcm14217727)
Supplement: Supplementary file 1 [file jcm-14-07727-s001.zip › jcm-3877652-supplementary.pdf]

Journal of
Clinical Medicine

MDPI

Supplementary Materials

Table S1. PRISMA 2020 Checklist.

(Note: This manuscript is a narrative review. The PRISMA checklist is provided for transparency regarding the review's structure and reporting, with items specific to systematic review methodology marked as "Not Applicable" (N/A).)

**Section and Topic**	**Item #**	**Checklist Item**	**Location Where Reported**
**TITLE**			
Title	1	Identify the report as a systematic review.	N/A (Narrative Review)
**ABSTRACT**			
Abstract	2	Provide a structured summary including, as applicable: background; objectives; data sources; study eligibility criteria, participants, and interventions; study appraisal and synthesis methods; results; limitations; conclusions and implications of key findings; systematic review registration number.	Abstract
**INTRODUCTION**			
Rationale	3	Describe the rationale for the review in the context of what is already known.	Introduction
Objectives	4	Provide an explicit statement of the question(s) the review addresses in terms of population, interventions, comparators, and outcomes (PICO).	Introduction
**METHODS**			
Eligibility criteria	5	Specify the inclusion and exclusion criteria for the review and how studies were grouped for the syntheses.	N/A
Information sources	6	Specify all databases, registers, websites, organizations, reference lists, and other sources searched or consulted to identify studies. Specify the date when each source was last searched or consulted.	N/A
Search strategy	7	Present the full search strategies for all databases, registers, and websites, including any filters and limits used.	N/A
Selection process	8	Specify the methods used to decide whether a study met the inclusion criteria of the review, including how many reviewers screened each record and each report retrieved, whether they worked independently, and if applicable, details of automation tools used in the process.	N/A
Data collection process	9	Specify the methods used to collect data from reports, including how many reviewers collected data from each report, whether they worked independently, any processes	N/A

Section and Topic	Item #	Checklist Item	Location Where Reported
		for obtaining or confirming data from study investigators, and if applicable, details of automation tools used in the process.	
Data items	10	List and define all outcomes for which data were sought. Specify whether outcome data were sought, and if so, the methods used to decide which results to collect.	N/A
Study risk of bias assessment	11	Specify the methods used to assess risk of bias in the included studies, including details of the tool(s) used, how many reviewers assessed each study and whether they worked independently, and if applicable, details of automation tools used in the process.	N/A
Effect measures	12	Specify for each outcome the effect measure(s) (such as risk ratio, mean difference) used in the synthesis or presentation of results.	N/A
Synthesis methods	13	Describe the processes used to decide which studies were eligible for each synthesis (such as tabulating the intervention characteristics and comparing against the planned groups for each synthesis (item 5)). Describe any methods required to prepare the data for presentation or synthesis, such as handling of missing summary statistics, or data conversions. Describe any methods used to tabulate or visually display results of individual studies and syntheses. Describe any methods used to synthesize results and provide a rationale for the choice(s). If meta-analysis was performed, describe the model(s), method(s) to identify the presence and extent of statistical heterogeneity, and software used. Describe any methods used to explore possible causes of heterogeneity among study results (such as subgroup analysis, meta-regression). Describe any sensitivity analyses conducted to assess the robustness of the synthesized results.	N/A
Reporting bias assessment	14	Describe any methods used to assess risk of bias due to missing results in a synthesis (arising from reporting biases).	N/A
Certainty assessment	15	Describe any methods used to assess certainty (or confidence) in the body of evidence for an outcome.	N/A
**RESULTS**			
Study selection	16	Describe the results of the search and selection process, from the number of records identified in the search to the	N/A

Section and Topic	Item #	Checklist Item	Location Where Reported
		number of studies included in the review, ideally using a flow diagram.	
Study characteristics	17	Cite each included study and present its characteristics.	Throughout Manuscript
Risk of bias in studies	18	Present assessments of risk of bias for each included study.	N/A
Results of individual studies	19	For all outcomes, present, for each study: (a) summary statistics for each group (where appropriate) and (b) an effect estimate and its precision (such as confidence/credible interval), ideally using structured tables or plots.	Throughout Manuscript
Results of syntheses	20	For each synthesis, present results of all statistical syntheses performed. If meta-analysis was done, present for each the summary estimate and its precision. Present results of all investigations of possible causes of heterogeneity among study results. Present results of all sensitivity analyses conducted.	N/A
Reporting biases	21	Present assessments of risk of bias due to missing results (arising from reporting biases) for each synthesis assessed.	N/A
Certainty of evidence	22	For each outcome assessed, present the certainty (or confidence) in the body of evidence.	N/A
**DISCUSSION**			
Discussion	23	Provide a general interpretation of the results in the context of other evidence. Discuss any limitations of the evidence included in the review. Discuss any limitations of the review processes used. Discuss implications of the results for practice, policy, and future research.	Discussion/Conclusion
**OTHER INFORMATION**			
Registration and protocol	24	Provide registration information for the review, including register name and registration number, or state that the review was not registered. Indicate where the review protocol can be accessed, or state that a protocol was not prepared.	N/A
Support	25	Specify the sources of financial or non-financial support for the review, and the role of the funders or sponsors in the review.	Source of Funding
Competing interests	26	Declare any competing interests of review authors.	Conflicts of Interest
Availability of data, code and other materials	27	Report which of the following are publicly available and where they can be found: template data collection forms; data extracted from included studies; data used for all	Transparency Statement

Section and Topic	Item #	Checklist Item	Location Where Reported
		analyses; analytic code; any other materials used in the review.	
